# Trends in psychotropic drug use in nursing home residents with dementia and their association with regulatory changes: A routine care data analysis from 2016 to 2023

**DOI:** 10.1371/journal.pone.0361903

**Published:** 2026-11-04

**Authors:** Charlotte A. W. Albers, Fenne Wouters, Martine C. de Bruijne, Martin Smalbrugge, Karlijn J. Joling

**Affiliations:** 1 Amsterdam UMC location Vrije Universiteit Amsterdam, Department of Medicine for Older People, Amsterdam, The Netherlands; 2 Amsterdam Public Health, Aging and Later Life, Amsterdam, The Netherlands; 3 Amsterdam Public Health, Methodology, Amsterdam, The Netherlands; 4 Amsterdam UMC location Vrije Universiteit Amsterdam, Department of Public and Occupational Health, Amsterdam, The Netherlands; 5 Amsterdam Public Health, Quality of Care, Amsterdam, The Netherlands; University of Thessaly Faculty of Medicine: Panepistemio Thessalias Tmema Iatrikes, GREECE

## Abstract

**Introduction:**

Non-pharmacological interventions are the first-choice treatment for managing neuropsychiatric symptoms in nursing home (NH) residents with dementia, yet psychotropic drugs (PDs) are frequently prescribed despite their limited effectiveness. In the current century, policies in various countries have increasingly emphasized the need for restraint in prescribing PDs, partly through amendments to guidelines and legislation. In the Netherlands, regulatory changes included the 2018 revision of the guideline for challenging behavior, the introduction of the Care and Coercion Act (CCA), and a new Dementia Care Standard in 2020. This study examined the prevalence of PD prescriptions (PDPs) from 2016 to 2023 among residents with dementia in Dutch NHs, and the course of the PDP trend in the context of regulatory changes, using routine care data.

**Methods:**

We analyzed routine care data from 11 NH organizations. Quarterly point prevalences were calculated for any PDP and five PD categories separately (antipsychotics, anxiolytics, hypnotics, antidepressants, anti-dementia drugs). We calculated confidence intervals to indicate temporal changes in PDP prevalence and assess the relationship with different reforms. We used interrupted time series (ITS) analysis to explore the association between regulatory changes and the PDP trend.

**Results:**

Data from 17,738 residents showed an overall decline in PDP prevalence from 48.3% in 2016 to 42.7% in 2023. Antidepressants and antipsychotics were the most frequently prescribed PDs. Antidepressants showed the largest absolute prevalence decline over time. Anti-dementia drugs showed the greatest relative decline. ITS showed a steeper declining trend after the combined introduction of the CCA and Dementia Care Standard in the second quarter of 2020, compared with the period after the guideline revision in 2018.

**Discussion:**

The PDP rate decreased over time but was still substantial. Regulatory changes, including new legislation and (clinical) guidelines, appear to have contributed to a further decline from Q2 2020 onwards. This decline suggests that policy and guideline interventions may meaningfully influence prescribing behavior, offering a promising lever for reducing potentially inappropriate psychotropic use in dementia care. Insight into the appropriateness of prescribing practices is essential to identify areas for further improvement.

## 1. Background

People with dementia and other neurocognitive disorders frequently exhibit neuropsychiatric symptoms (NPSs), such as agitation, apathy, or sleep disturbances [1]. These symptoms significantly impact individuals with dementia, their relatives, and care professionals [2]. Therefore, effective NPS management is essential.

Although most guidelines recommend non-pharmacological interventions as the first-line treatment and new research is being performed to find suitable interventions for NPSs [3], psychotropic drugs (PDs) are still often prescribed to manage challenging behaviors [4]. However, the evidence supporting the efficacy of PDs in treating NPSs in dementia is limited, and their use can result in severe adverse effects, such as an increased risk of falls or fractures [5–10]. Besides, people with dementia are more susceptible to polypharmacy and drug-drug interactions, and adverse drug events can decrease the patients’ quality of life and worsen their prognosis [11]. Therefore, it is not only important to reduce PD prescriptions (PDPs) overall, but also to reduce the number of different PDs a patient receives.

In the Netherlands, about 25% of people with dementia live in nursing homes (NHs) [12]. Several studies have assessed the prevalence of PDPs in NH residents with dementia. Across Western European NHs, PDs, especially antipsychotics and antidepressants, are widely prescribed, with antipsychotic use ranging from 12% to 59% and antidepressant use ranging from 19% to 68% [13]. In the Netherlands, Zuidema et al. [14] reported a stable PDP prevalence of approximately 60% from 2003 to 2011. However, a more recent analysis using baseline data from cohort studies and cluster-randomized controlled trials (RCTs) from the Netherlands noted a decrease from 63% in 2003 to 40% in 2018, with antipsychotics and antidepressants remaining the most commonly prescribed PDs [15].

In the current century, policies in various countries have increasingly emphasized the need for restraint in prescribing PDs, partly through amendments to guidelines and legislation. Already in 2005, the United States Food and Drug Administration (FDA) issued a public health advisory warning of fatal adverse events in patients with dementia treated with atypical antipsychotic drugs [16]. The United Kingdom developed a national dementia strategy in 2009 [17]. In Australia, the Royal Commission into Aged Care Quality and Safety, which ran from 2018 to 2021, introduced several aged care reforms, including targeting the inappropriate use of chemical restraint and the mandatory reporting on antipsychotic use [18,19]. More recently, the FDA wrote a report to address the inappropriate use of antipsychotic drugs in NHs [20].

In the Netherlands, long-term care (LTC) has also undergone substantial changes in recent years. First, in 2015, the Dutch government introduced a major LTC reform with the new Long-Term Care Act (Dutch: Wet Langdurige Zorg, WLZ). Most importantly, they initiated the reform to control spending growth and ensure the long term financial viability of LTC, as well as to improve its quality by making it more client-tailored. One pillar of the reform was shifting from residential to non-residential care [21], with residents admitted to NHs at more advanced stages of dementia, exhibiting more severe challenging behavior [22]. Second, the Dutch Association of Elderly Care Physicians (Dutch: Verenso) revised its guideline on challenging behavior in 2018 [23], providing explicit recommendations on PDPs for NH residents with dementia by placing more emphasis on non-pharmacological interventions and increased restraint in PDPs. Third, a new law concerning involuntary care for people with psychogeriatric syndromes was enacted in 2020, called the Care and Coercion Act (CCA) [24]. This legislation equates PDs to involuntary care if they are not prescribed in line with guideline recommendations for persons unable to consent, theoretically raising the threshold for prescribing PDs. Finally, in 2020, the Dementia Care Standard 2020 was launched as a revised version of the Dementia Care Standard 2013, alongside national campaigns by Alzheimer Nederland promoting minimal use of PDs and greater use of psychosocial interventions [25,26]. Given these changes over the last decade, it is important to know whether these initiatives reduced the prevalence of PDPs.

Recent attention has focused on reducing PDPs. However, the relationship between regulatory changes and PDP rates remains unclear. To address this gap, research using a large, unselected sample over an extended period is needed. Most previous studies on PDP prevalence in residents with dementia relied on clinical trial data [14,15] or cross-sectional designs, which limit insight into PDP patterns over time [27–32]. Studies using routine care data over extended periods were conducted less recently (2000–2013) [33,34]. Therefore, this study aims to assess the prevalence of PDPs among residents admitted to dementia special care units in Dutch NHs and the course of the PDP trend in the context of regulatory changes, using electronic prescription system (EPS) data from 2016 to 2023. Using routine care data allows us to evaluate real-world prescribing practices over time, reflecting usual clinical practice rather than research-driven data collection, across a large, unselected population that would be difficult to capture through prospective data collection alone. To reach this aim, we have formulated the following research question: What is the prevalence of PDPs among NH residents with dementia between 2016 and 2023, and are observed prevalences of PDPs associated with regulatory changes?

## 2. Materials and methods

This study used an observational design over a long period, using routine care data from electronic health records (EHRs) and an EPS for people residing in dementia special care units in NHs in the Netherlands.

### 2.1. Data sources

We conducted this research within the ‘Learning from Data’ (Dutch: Leren van Data) research program, which promotes the (re)use of routine care data in NHs [35]. Within this research program, we use pseudonymized data from the EHR Ysis and EPS Medimo for scientific research. Of the 27 organizations that granted permission to share data from both EHR Ysis and EPS Medimo for this research, 11 used both systems at the start and throughout the study period; therefore, we included data from NH residents in these 11 organizations in the study sample.

### 2.2. Study sample

We included NH residents residing in a dementia special care unit for at least one day between 1 January 2016 and 31 March 2023. Most people with dementia or similar psychogeriatric syndromes residing in NHs are admitted to dementia special care units [36]. Therefore, we included only residents of dementia special care units and excluded those who received geriatric rehabilitation or somatic care. The dementia special care units included residents of four types of care: chronic psychogeriatric care, gerontopsychiatric care, Huntington care, and Korsakoff care. Residents in gerontopsychiatric, Huntington, and Korsakoff care units were included alongside people with chronic psychogeriatric care needs, as these groups share a comparable profile of chronic cognitive and behavioral impairment and require the same intensity of long-term, 24-hour supervised care. Some of the residents therefore do not have a dementia diagnosis. In addition, some dementia diagnoses may not have been recorded in the EHR. We excluded data from residents with an unexpected age (<18 or >110 years) (n = 1) or a negative length of stay (n = 22) (<0.01%).

### 2.3. Data collection

We extracted data from the EPS and EHR for analysis between January 2016 and March 2023. We accessed the data on 14 February 2024. We retrieved data on age, sex, type of care, date of admission, (when applicable) date of deregistration or death, and organizational ID from the EHR. Furthermore, the presence of various medical conditions was assessed, including COPD, heart failure, other cardiovascular diseases without heart failure, stroke, diabetes, reduced kidney function, liver disease, dementia, Parkinson’s disease, Korsakoff, Huntington’s disease, other chronic progressive neurological diseases, delirium, cancer or leukemia, and indication of palliative care or terminal/end-stage disease. We derived the conditions from the medical history free-text field in the EHR using a query with the most common disease names and synonyms to extract the mentioned diagnoses [37]. A researcher and elderly care physician developed the query, and a medical informatician refined it. It is based on the comorbidities that are used to calculate the Charlson Comorbidity Index [38]. To improve the query, elderly care physicians (in training) tested it using an iterative process. Residents whose medical history consisted completely of conditions that we did not include in our comorbidity list were classified as having ‘other conditions only’ [39].

Information on medication prescriptions, such as drug ‘Anatomical Therapeutic Chemical’ (ATC) code, start and stop dates, administration frequency, dosing, and whether it was a regular or ‘as needed’ (pro re nata, PRN) prescription, was extracted from the EPS. Based on the first four alphanumeric characters of the drug ATC code, PDPs can be divided into main categories: antidepressants (N06A), anxiolytics (N05B), hypnotics (N05C), antipsychotics (N05A), and anti-dementia drugs (N06D) (World Health Organization, 2024). Anticonvulsants (N03A) were excluded from this study because they are frequently prescribed for reasons other than NPS, such as epilepsy and neuropathic pain [40]. Baseline variables such as year of birth and sex had no missing values, just as variables on medication prescriptions. When information to define the type of care and admission period was missing, we excluded these residents (almost 40% of the data rows). From previous work, we know that people without an admission goal are mostly those residing in the community or receiving short-term care and were therefore probably not part of our study population.

### 2.4. Prevalence of psychotropic drugs

The point prevalence of PDPs was calculated by dividing the number of residents with at least one PDP on the first day of each quarter of the year by the number of residents residing in a participating NH in a dementia special care unit on that day, from January 1, 2016, to March 31, 2023. Quarterly point prevalence of PDPs was calculated overall (i.e., a prescription in at least one of the PD categories) and for the five main ATC categories separately. In the analysis, we distinguished between regular and PRN prescriptions. To assess psychotropic polypharmacy, we calculated the number of different categories from which a resident had a prescription. Based on the five main ATC categories, residents with a PDP could have prescriptions in at least one and up to five different categories. To assess inter-organizational differences, we also calculated the overall quarterly point prevalence of regular PDPs per organization. To assess whether the exclusion of anticonvulsants may have obscured substitution effects, we calculated the quarterly prevalence of anticonvulsant use separately, using the same methods and reported the outcomes in the supporting information (S5 Table).

### 2.5. Data analysis

We used IBM SPSS Statistics 28 [41], R and RStudio version 4.4.3 [42] to analyze the data, and figures were produced using the package ggplot2 [43] and GraphPad Prism version 10.6.0 [44]. We used descriptive statistics to assess the quarterly point prevalence of PDPs among NH residents admitted to dementia special care units from 2016 to the first quarter of 2023. To assess temporal changes in prevalence and course of the PDP trend in the context of regulatory changes, we calculated Wilson score 95% confidence intervals (CIs) for each quarter. We compared them to three quarters matching the regulatory changes: Q1 2016 for the first available quarter after the introduction of the new Long-Term Care Act, Q1 2018 for the revision of the guideline on challenging behavior, and Q2 2020 for the combined introduction of the CCA and the Dementia Care Standard 2020. When looking only at regular PDPs, the reference values are Q1 2016: 0.483 (95% CI, 0.467 to 0.499); Q1 2018: 0.476 (95% CI, 0.460 to 0.491); and Q2 2020: 0.452 (95% CI, 0.437 to 0.467). When including PRN PDPs, the reference values are Q1 2016 (0.193; 95% CI, 0.181 to 0.206), Q1 2018 (0.215; 95% CI, 0.203 to 0.228), and Q2 2020 (0.215; 95% CI, 0.202 to 0.227). We used non-overlapping 95% CIs as a conservative indicator of differences between quarters. As these comparisons were exploratory, no adjustment for multiple comparisons was applied. We used interrupted time series (ITS) analysis to explore whether changes in the prevalence trend of PDPs coincided with regulatory changes. The 2015 Long-Term Care Act preceded the study period and was not modeled. We combined the introduction of the CCA (Q1 2020) and the Dementia Care Standard 2020 (Q2 2020) into a single period, resulting in three periods: baseline (2016 Q1-2017 Q4), post-guideline revision (2018 Q1-2020 Q1), and post-dementia care standard/CCA (2020 Q2 onward). We first compared mean prevalence between periods. We then used segmented linear regression, including a dummy-coded period variable, a continuous quarter variable, and their interaction, to test whether the linear trend across quarters differed between periods, allowing effects to be evaluated relative to both baseline and the immediately preceding period.

### 2.6. Ethics

The Medical Ethics Committee of the VU University Medical Center determined that the Medical Research Involving Human Subjects Act (Dutch: WMO) did not apply to this study and granted a waiver for ethics approval (reference number: METc VUmc 2016.516 and 2022.0076). The authors did not have access to information that could identify individual participants during or after data collection.

We used a no-objection (opt-out) procedure, in which residents of NHs were informed by their healthcare organization about the use of their pseudonymized data and could object to its use for scientific studies. We excluded data from residents who objected (<1% of residents).

## 3. Results

### 3.1. Study sample

This study included 17,738 residents from 11 Dutch NH organizations. Table 1 presents the baseline characteristics. Most of the residents resided on a department for chronic psychogeriatric care (97.5%). Most residents were female (64.6%), and the median age at admission was 84.0 years (IQR 10.0 years). The median length of stay until deregistration or death was 18.4 months (IQR 31.3 months). 87.1% of the residents had a recorded dementia diagnosis in their medical history.

**Table 1:** Baseline characteristics.

	Total residents (n = 17,738)
**Female, n (% of N)**	11,457 (64.6)
**Median age at admission, years (IQR)**	84.0 (10.0)
**Median age at admission by year of admission, years (n, IQR)**	
2010	80.0 (147, 12.0)
2011	81.0 (252, 9.0)
2012	82.0 (443, 10.0)
2013	83.0 (690, 10.0)
2014	83.0 (828, 9.75)
2015	84.0 (1403, 10.0)
2016	85.0 (1772, 9.0)
2017	85.0 (1688, 10.0)
2018	85.0 (1778, 10.0)
2019	85.0 (1760, 10.0)
2020	85.0 (2110, 10.0)
2021	85.0 (2072, 11.0)
2022	84.0 (2170, 14.0)
2023	85.0 (625, 10.0)
**Type of nursing home ward, n (% of N)**	
Chronic psychogeriatric care	17,296 (97.5)
Gerontopsychiatry	142 (0.8)
Huntington	105 (0.6)
Korsakoff	195 (1.1)
**Median length of stay until deregistration or death, months (n, IQR)**	18.4 (14.401, 31.3)
**Median length of stay until deregistration, death or extraction date by year of admission, months (n, IQR)**	
2010	87.0 (147, 27.0)
2011	73.0 (252, 23.1)
2012	59.6 (443, 29.3)
2013	49.9 (690, 29.6)
2014	38.4 (828, 30.9)
2015	28.2 (1403, 30.9)
2016	18.4 (1772, 32.4)
2017	17.6 (1688, 30.6)
2018	18.8 (1778, 26.3)
2019	14.7 (1760, 23.8)
2020	11.3 (2110, 20.9)
2021	9.9 (2072, 15.9)
2022	6.2 (2170, 9.7)
2023	3.5 (625, 6.2)
**Conditions in medical history, n (% of N)**	
COPD	3,155 (17.8)
Heart failure	2,954 (16.7)
Other cardiovascular diseases without heart failure	3,195 (18.0)
Stroke	4,574 (25.8)
Diabetes	4,568 (25.8)
Reduced kidney function	3,423 (19.3)
Liver Disease	448 (2.5)
Dementia	15,448 (87.1)
Parkinson’s disease	664 (3.7)
Korsakoff	474 (2.7)
Huntington’s disease	127 (0.7)
Other chronic progressive neurological diseases	1,849 (10.4)
Delirium	2,355 (13.3)
Cancer or Leukemia	5,342 (30.1)
Indication palliative care or terminal/end-stage disease	145 (0.8)
Depression	2782 (15.7)
Anxiety disorder	1345 (7.6)
Other conditions only*	136 (0.8)
No medical history recorded	81 (0.5)
Charlson Comorbidity Index (CCI)**, median (IQR)	3 (2-5)
0	324 (2)
1–2	6358 (35.8)
3–4	6206 (35.0)
>= 5	4741 (26.7)

* The medical history does not include any of the aforementioned conditions.

** CCI was calculated using the original Charlson weights, based on diagnoses recorded in the EHR at baseline. Higher scores indicate greater comorbidity burden.

### 3.2. Analyses including only regular prescriptions

#### 3.2.1. Psychotropic drug prescription prevalences in total.

9,059 residents had a regular PDP in at least one of the quarters. The PDP-prevalence results of regular PDPs are presented both in total (for all five PD categories together, having at least one PDP) (Fig 1, S1 Table) and in five individual categories separately (Fig 1, S2 Table). Overall, we observed a decline in PDP prevalence from the first quarter of 2016 to the first quarter of 2023. In the first quarter of 2016, prevalence was 48.3%, which decreased, then increased slightly until the first quarter of 2018 (47.6%) after which it declined slightly but remained mostly stable. The declining trend strengthens from Q4 2019 onwards and the PDPs prevalence subsequently decreased again to 42.7% in the first quarter of 2023. Over seven years, the prevalence of PDPs changed by −5.6 percentage points (95% CI [−7.7, −3.5]) in absolute terms and −11.6% (95% CI [−15.6%, −7.3%]) relative to baseline.

**Fig 1:**
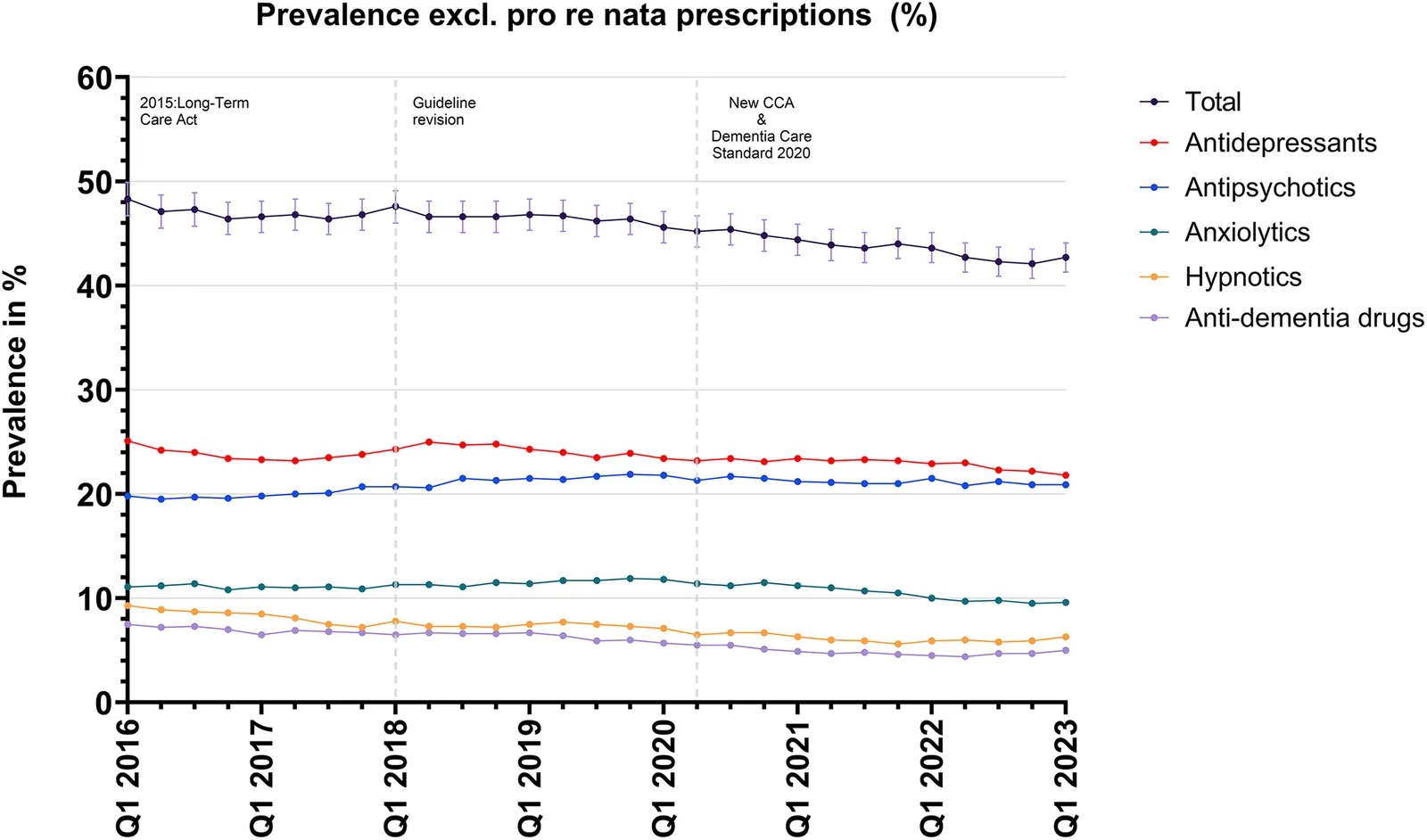
Prevalence of regular PDPs, overall and for each of the five drug categories. Excluding PRN prescriptions.

To assess temporal changes in overall PDP prevalence and regular PDP prevalence across quarters and regulatory changes, we examined the 95% CIs and compared them with the three reference periods (Fig 1, S1 Table).

Compared with Q1 2016, all quarters from Q4 2020 onward showed non-overlapping CIs, indicating a sustained decrease in overall PDP prevalence beginning in late 2020. Compared with Q1 2018, a significant decrease starts in Q1 2021. Finally, compared with Q1 2020, non-overlap is clearest from Q3 2022 onwards.

Mean prescribing prevalence was 46.96% (95% CI [46.35, 47.58]) during the baseline period. No significant difference was observed following the guideline revision (46.57%, mean difference = −0.40, 95% CI [−1.24, 0.45], p = .343). Mean prevalence was significantly lower following the combined introduction of the Dementia Care Standard 2020 and the CCA (43.72%) compared with both baseline (mean difference = −3.24, 95% CI [−4.03, −2.45], p < .001) and the post-guideline revision period (mean difference = −2.84, 95% CI [−3.61, −2.08], p < .001).

ITS, accounting for the linear trend across quarters, confirmed a significant decline in prescribing prevalence before any regulatory change (difference in trend = −0.18 per quarter, 95% CI [−0.31, −0.06], p = .005). No significant change in trend was observed following the guideline revision relative to baseline (difference in trend = 0.03, 95% CI [−0.13, 0.19], p = .729). After the combined introduction of the Dementia Care Standard 2020 and the CCA, the rate of decline became significantly steeper than the post-guideline trend (difference in trend = −0.14 per quarter, 95% CI [−0.26, −0.02], p = .029).

#### 3.2.2. Psychotropic drug prescription prevalences per category.

Antidepressants and antipsychotics were the most commonly prescribed PDs. S2 Table describes all quarterly prevalences and 95% CIs. The prescription rate of antidepressants followed a similar declining trend over time as the overall PDP prevalence, showing an absolute decrease of 3.2 percentage points (95% CI [−5.1, −1.4]) and a relative decrease of 12.9% (95% CI [−19.4%, −5.9%]). Together with antidepressants, both hypnotics (−2.9 percentage points, 95% CI [−4.1, −1.8]) and anti-dementia drugs (−2.5 percentage points, 95% CI [−3.5, −1.5]) showed the greatest absolute decline. Hypnotics and anti-dementia drugs also showed the greatest relative decline, with −31.7% (95% CI [−41.2%, −20.8%]) and −33.3% (95% CI [−43.7%, −21.1%]), respectively. Anxiolytics showed the smallest absolute decline (−1.5 percentage points, 95% CI [−2.8, −0.2]) but a relative decline of 13.4% (95% CI [−23.7%, −1.8%]). Antipsychotic prescription rates increased by 1.1 percentage points (95% CI [−0.6, 2.9]) in absolute terms and by 5.7% (95% CI [−2.9%, + 15.1%]) relative to baseline, but this increase was not statistically significant.

The prevalence of anticonvulsant use remained relatively stable over the study period, ranging from 4.3% to 5.5% (S5 Table).

#### 3.2.3. Psychotropic polypharmacy.

Although more than half of the population did not have a PDP in one of the five ATC drug categories, some residents received PDs from multiple categories (Fig 2). Most residents with a PDP (29.2% in Q1 2016 to 26.2% in Q1 2023) had prescriptions in only one of the five PD categories. Fewer people in the study sample (14.2% in Q1 2016 to 12.4% in Q1 2023) had PDPs across two of the five ATC drug categories. When combining two types of PDs, residents usually receive an antipsychotic and antidepressant drug. The percentages of residents with PDPs across three of five PD categories ranged from 4.2% in 2016 to 3.5% in 2023. Finally, fewer than 1% of residents had PDPs in four or all five categories. Over time, the percentage of residents with a prescription in at least two concurrent PD categories decreased from 19.0% in the first quarter of 2016 to 16.5% in the first quarter of 2023 (absolute difference −2.6 percentage points, 95% CI [−4.2, −0.9]; relative difference −13.5%, 95% CI [−21.1%, −5.1%]).

**Fig 2:**
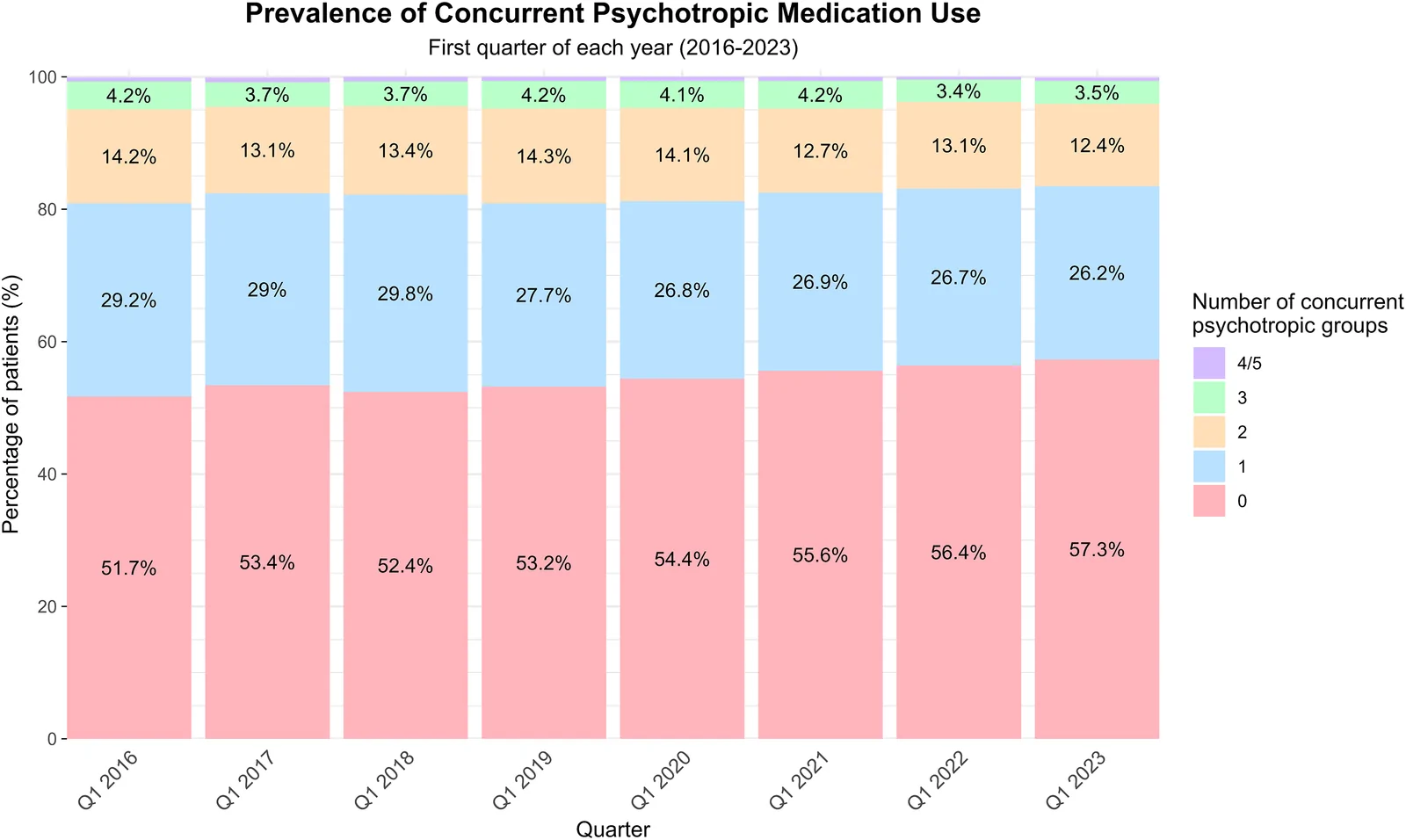
Prevalence of concurrent psychotropic medication use.

#### 3.2.4. Interorganizational differences.

Differences were observed between the 11 participating organizations (Fig 3). In the first quarter of 2016, prevalence estimates for regular PDPs ranged from 44.4% to 58.1%, with one organization reporting a much lower prevalence of 9.7%. In the first quarter of 2023, prevalence ranged from 34.4% to 65.8%, with the same organization again showing a much lower value (6.6%).

**Fig 3:**
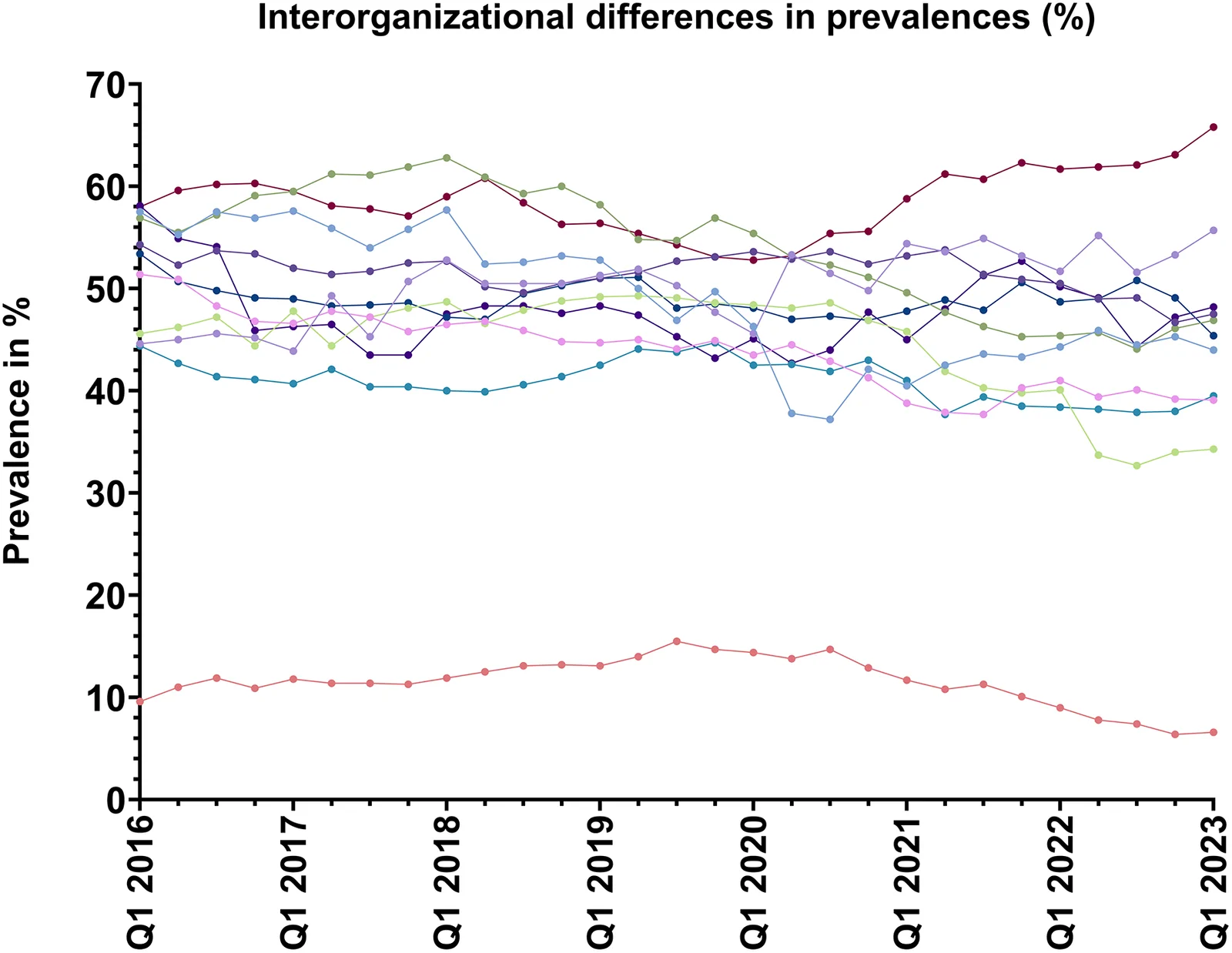
Prevalence of regular PDPs overall, per organization.

To test the robustness of the declining trend, we performed a sensitivity analysis. We excluded the outlier organization, resulting in a prevalence of 58.3% in Q1 2016 and 50.4% in Q1 2023. This reflects an absolute decline of 7.9% and a relative decline of 13.6%. The outlier organization is therefore not driving the overall declining trend.

### 3.3. Prevalence of only pro re nata prescriptions

5,556 residents received a PRN PDP in at least one of the quarters. Overall, we observed a relatively stable prevalence trend over time (Fig 4, S3 Table). The overall PRN PDP rate started at 19.3% in the first quarter of 2016, increased to a maximum of 22.6% in the first quarter of 2021, and then decreased again to 18.9% in the first quarter of 2023. This reflected an absolute decline of 0.4 percentage points (95% CI [−2.1, 1.3]) and a relative decline of 1.9% (95% CI [−10.2%, 7.2%]).

**Fig 4:**
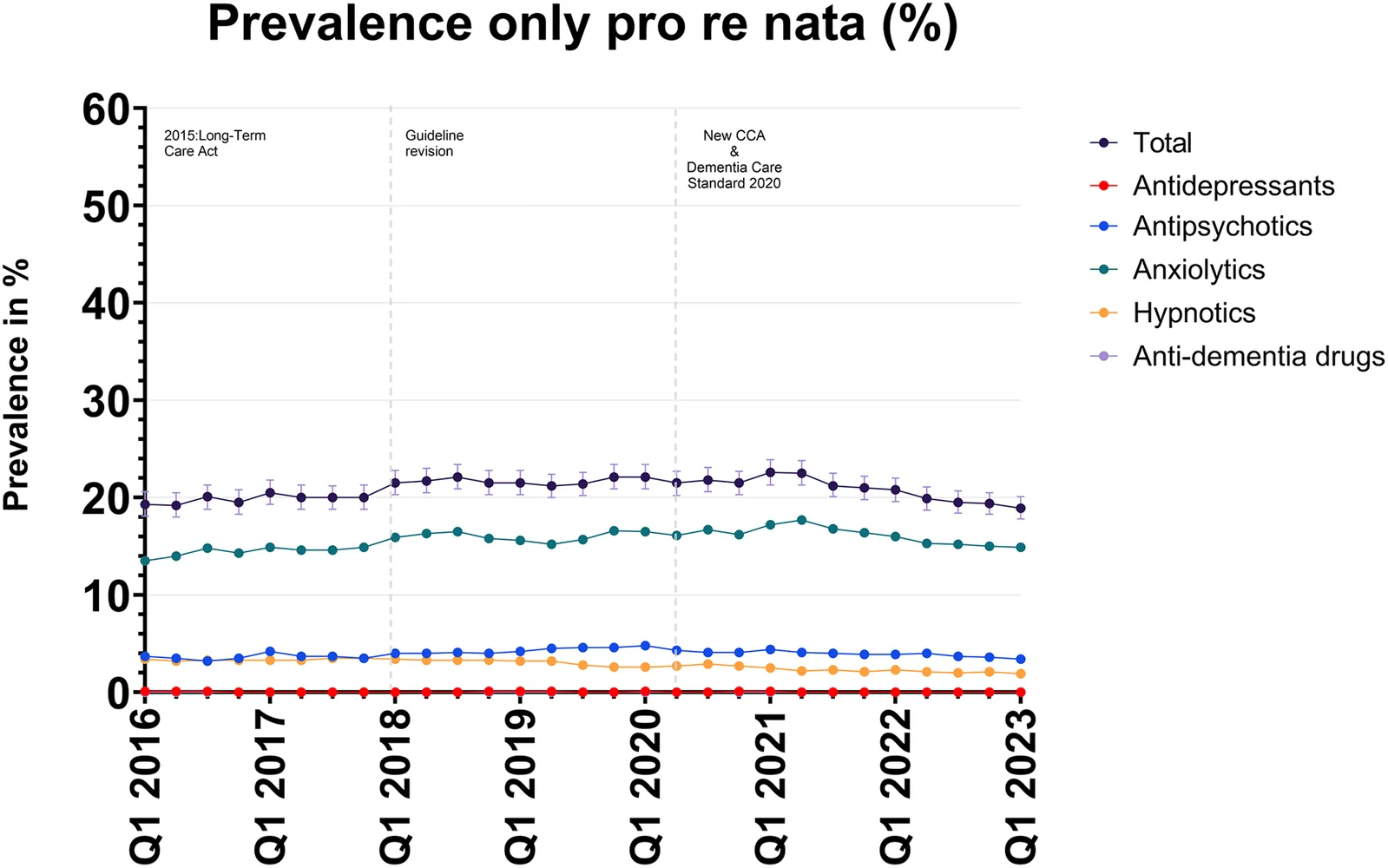
Prevalence of PDPs, overall and for each of the five drug categories, only including PRN prescriptions.

Of all 5 categories, anxiolytics were most often prescribed PRN (Fig 4, S4 Table). With a prevalence of 13.5% in the first quarter of 2016 and 14.9% in the first quarter of 2023, prescriptions increased slightly and non-significantly; absolute change of +1.4 percentage points (95% CI [−0.1, 2.9]) and a relative change of +10.3% (95% CI [−0.8%, 22.7%]). Antipsychotics and hypnotics showed a similar prescription trend to each other, with a rate below 5%. Finally, antidepressants and anti-dementia drugs were (almost) never prescribed PRN.

Compared with Q1 2016 as the reference period, there was no clear trend (Fig 4, S3 Table), with some sporadic non-overlapping confidence intervals. Compared with Q1 2018, only the CI for Q1 2023 shows no overlap. Compared with Q1 2020, confidence intervals are non-overlapping from Q3 2022 onwards.

## 4. Discussion

In dementia special care units of NHs in the Netherlands, the prevalence of PDPs decreased from 48.3% in 2016 to 42.7% in 2023. Antidepressants and antipsychotics were the most commonly prescribed PDs. Antidepressants showed the largest absolute decline in prevalence, whereas the greatest relative decline was observed in anti-dementia drugs and hypnotics. Antipsychotics were the only drug category for which a small increase in prescriptions was observed. For regular PDPs, we observed a steeper declining trend after the combined introduction of the CCA and the Dementia Care Standard 2020.

As described in the introduction, several countries have increasingly emphasized a more conservative approach to prescribing PDs. In the Netherlands, several regulatory changes may together have influenced the observed trend. On the one hand, the 2015 LTC reform led to fewer NH admissions in the first year after the reform, a higher age at admission, and a shorter average survival time (Table 1), suggesting that residents require more intensive care and enter the NH in a more advanced stage of dementia [45], in which NPS are more prevalent [1]. Staff shortages and associated workload pressure may also influence psychotropic prescribing, with inadequate staffing potentially prompting on-site requests to initiate PDs [46,47]. On the other hand, the revised guideline for challenging behavior [23], the CCA [24], the Dementia Care Standard 2020, and increased attention to de-implementation to avoid continued use [48] may have reduced PDPs. The steeper declining trend in the period after the combined introduction of the CCA and the Dementia Care Standard 2020 could indicate that the 2020 regulations may have accelerated an already declining trend.

The introduction of the CCA and Dementia Care Standard 2020 coincided with the COVID-19 pandemic, which may have affected prescribing independently. Evidence on the pandemic’s impact on PD prescribing in NHs is mixed: a Dutch study found that PD use remained stable during the first wave (Sizoo, 2022), whereas studies from Canada [49,50] and Spain [51] reported increases, potentially linked to disruptions in staffing, support, and services.

### 4.1. Prevalence of regular psychotropic drug prescriptions

Comparison with other (international) studies shows varying results. A recent cross-sectional study from Belgium (2023) found a total PDP prevalence of 68.6%, which is much higher than ours, possibly due to their cross-sectional design with a one-month data collection period and the inclusion of other medication types, such as anticonvulsants, which we excluded. Compared with NHs in the United States, results are inconsistent: Oregon Memory Care Units reported a 90-day antipsychotic prevalence of 43% [52], whereas a cross-sectional study from Texas found much lower antipsychotic prescription rates in NH residents with dementia, strongly decreasing from 22.1% in 2015 to 12.4% in 2020 [53]. These differences reflect how LTC is organized, financed, and regulated across states and countries [54,55].

Regarding the most recent Dutch research, Du et al. [15], examined the PDP prevalence between 2003 and 2018 using data from previous cross-sectional, cohort, and RCT studies. We found a continuing decline beyond their observed period, although our prevalences were slightly higher overall: 47.6% in the first quarter of 2018 versus 40.4% in 2018. Similar to Du et al. we found that antipsychotics and antidepressants were the most frequently prescribed PDs. An important methodological difference is that Du et al. primarily relied on data from RCTs, which offer high internal validity, but are also prone to selection bias due to strict inclusion and exclusion criteria, limiting generalizability to the overall NH population. In addition, Du et al. adjusted for dementia and NPS severity, which may partly explain the differences with our results.

### 4.2. Categories showing the greatest absolute and relative decline

The marked decline in antidepressant prevalence is noteworthy, as antidepressants are the most commonly prescribed PD, while evidence supporting their effectiveness for NPSs in Alzheimer’s dementia is limited [56]. Our results show a favorable decline, in line with Du et al. [15], possibly reflecting improved medication review practices, although targeted efforts to review antidepressants during admission and ongoing care could further reduce inappropriate use. Although anti-dementia drugs were prescribed to a small group of residents, the substantial relative decline of 33% across the study period is also a favorable trend: over the years, guidelines have become more reluctant to prescribe anti-dementia drugs to people with dementia at an advanced stage showing agitated behavior, and more attention is being paid to deprescribing as there is uncertainty regarding benefits and risks regarding anti-dementia drugs [23,57,58].

### 4.3. Increase in antipsychotic prescription prevalence

Contrary to the favorable decline in most PD categories, we found a slight increase of 1% in the prevalence of antipsychotic prescriptions. The prevalence of around 20% to 21% is in line with other research in Dutch NH residents with dementia during the first wave of COVID-19, which found an antipsychotic prescription prevalence of 21.0% to 22.9% from February to August 2020 [59]. This increase might be explained by the more advanced stages of dementia at which people enter the NH, as discussed above [22], because antipsychotics can be appropriate when behavioral symptoms are severe [23]. In addition, antipsychotics are increasingly prescribed off-label among elderly individuals, particularly those with dementia, which may have contributed to the overall increase in prescriptions [60].

### 4.4. Interorganizational differences

In line with the interorganizational differences we found in PDP prevalences, Kleijer et al. reported [61] considerable variation in antipsychotic use between Dutch NHs (5%–52%). NHs with a high prevalence of antipsychotic use were often large, situated in urban communities, and scored below average on staffing, personal care, and recreational activities. Smeets et al. [32] found that several non-resident-related factors could influence PDPs: higher emotional distress in nurses due to NPS could lead to a higher number of PDPs, whereas higher availability of physicians led to fewer antipsychotic prescriptions. These findings suggest distinct prescribing practices across organizations. Benchmarking NH data against other organizations might improve quality of care within the NH and reduce differences in quality of care between NHs [62].

### 4.5. Pro re nata prescriptions

When including only PRN PDPs, we mainly found a high prevalence in the anxiolytics group. Anxiolytics are often prescribed to reduce anxiety, in case of sleeping disorders (due to anxiety), or to temporarily sedate a person in case of an acute crisis [23]. As these situations generally occur sporadically, anxiolytics are more often prescribed PRN than the other categories. The combination of steadily declining regular PDP rates and stable PRN PDPs is a favorable trend that might indicate that prescriptions are not being substituted, although substitution at the resident level cannot be ruled out.

### 4.6. Strengths and limitations

A major strength of this study is the use of routine care data from the EPS and EHRs of Dutch NH organizations over a long period and a large sample of residents, providing insight into the influence of regulatory changes. Because these data reflect usual clinical practice rather than research-driven data collection, they realistically represent PDPs in Dutch NHs. Including 11 NH organizations yielded a relatively large sample of nearly 18,000 NH residents with baseline characteristics comparable to those of residents with dementia in Dutch NHs, enhancing the external validity of the findings [45]. In addition, the retrospective nature of the data reduces the likelihood that prescribing behavior was influenced by awareness of the study, further supporting the objectivity of the outcomes. Bias could have been introduced by including only NHs that permitted the use of pseudonymized data for scientific research, which are likely more interested in quality of care and therefore more inclined to follow guideline recommendations.

One of the main limitations of this study is that routinely recorded care data are not primarily documented for research purposes and may therefore lack information necessary for accurate classification and analysis. Although prescription data from the EPS were generally standardized and structured, prescription indications and data on non-pharmacological interventions were missing. Consequently, we could not determine which PDs were prescribed for challenging behavior, whether the prescriptions were clinically appropriate, or whether reductions in PDPs were accompanied by increased use of non-pharmacological approaches. For this reason, we also excluded anticonvulsants from the analysis.

Moreover, as PDs may have indications other than dementia and NPS (e.g., schizophrenia, bipolar disorder, depressive disorder, anxiety disorder), our prevalence estimates may overestimate PDPs for NPS in NH residents with dementia. Conversely, because anticonvulsants were excluded but are sometimes used off-label for NPS, our estimates may underestimate PDPs for NPS. However, the stable prevalence of anticonvulsant use over time suggests that substitution effects were limited (S5 Table). As data on dementia severity and NPS severity were also unavailable, we could not determine whether prescribing trends reflected changes in prescribing appropriateness or changes in the population’s clinical characteristics over time, or both. Furthermore, because our data are prescription data rather than administration data, we cannot define how often residents actually used their PRN medication. However, because medication administration must be signed off in Dutch NHs, we can reasonably assume that scheduled medication was actually administered. In addition, relevant patient characteristics, such as enrolment dates or the type of NH department, were in about 40% of the data rows missing due to recording issues, requiring the exclusion of these data to ensure reliable estimates. Although measuring point prevalence on the first day of each quarter ensured that all residents had an equal observation duration, it may have missed within-quarter changes in prescribing. Also, as an ITS analysis was performed only for overall regular PDPs, the observed differences in individual drug categories and PRN prescriptions were based on comparisons between two time points. These comparisons do not account for pre-existing trends and could therefore not be related to specific regulatory changes. We did not adjust for comorbidity burden in the ITS model. And finally, because the introduction of the CCA and Dementia Care Standard 2020 coincided with the COVID-19 pandemic, which may also have affected prescribing, the effects of these events could not be disentangled.

## 5. Conclusions and implication

While the declining trend in PDPs among Dutch NH residents in dementia special care units is encouraging, the increase in antipsychotic prescriptions and the persistent high rates across psychotropic categories indicate that continued effort is required to further align daily practice with evidence-based guidelines and optimize care quality for this vulnerable population. Regulatory changes, especially the combined introduction of the CCA and Dementia Care Standard 2020, may have contributed to the declining prevalence trend of PDPs. However, other factors, such as COVID-19, changes in staffing levels, and increased workload pressure, may also have influenced this trend, and because of the observational design, strong conclusions about causal relationships cannot be drawn. Improved de-implementation of revised guideline components and greater attention to non-pharmacological interventions could contribute to lower PDP rates. From a policy perspective, the observed interorganizational variation suggests that quality standards, implementation support, and benchmarking could help elevate performance across all facilities.

Future research could monitor whether the decline in PDP prevalence continues and whether PD prescribing aligns with guideline recommendations. Also, future studies could explore how the doses of PDPs have changed over time. Additionally, identifying barriers and facilitators to further PDP reduction, particularly for antipsychotics and in relation to NH characteristics, would inform targeted quality improvement interventions.

## Supporting information

S1 Table Prevalence of PDPs in total with 95% confidence intervals, including only regular prescriptions. (DOCX)

S2 Table Prevalence of PDPs in total and per category, including only regular prescriptions. (DOCX)

S3 Table Prevalence of PDPs in total with 95% confidence intervals, including only pro re nata prescriptions. (DOCX)

S4 Table Prevalence of PDPs in total and per category, including only pro re nata prescriptions. (DOCX)

S5 Table Prevalence of anti-convulsant prescriptions in total with 95% confidence intervals, including only regular prescriptions. (DOCX)
