# Supplementary material for: Trends in psychotropic drug use in nursing home residents with dementia and their association with regulatory changes: A routine care data analysis from 2016 to 2023
Source: PLoS One. 2026 Nov 4;21(11):e0361903. doi: 10.1371/journal.pone.0361903 (PMC13734491; doi:10.1371/journal.pone.0361903)
Supplement: Supplementary file 1 [file pone.0361903.s001.docx]

**S1 Table. Prevalence of PDPs in total with 95% confidence intervals, including only regular prescriptions.**

| Year | Quartile | Sample | Proportion | 95% CI |
| --- | --- | --- | --- | --- |
| 2016 | Q1 | 3765 | 48.3% | [46.7%, 49.9%] |
|  | Q2 | 3755 | 47.1% | [45.5%, 48.7%] |
|  | Q3 | 3904 | 47.3% | [45.7%, 48.9%] |
|  | Q4 | 3915 | 46.4% | [44.9%, 48.0%] |
| 2017 | Q1 | 4028 | 46.6% | [45.1%, 48.1%] |
|  | Q2 | 4042 | 46.8% | [45.3%, 48.3%] |
|  | Q3 | 4070 | 46.4% | [44.9%, 47.9%] |
|  | Q4 | 4066 | 46.8% | [45.3%, 48.3%] |
| 2018 | Q1 | 4024 | 47.6% | [46.0%, 49.1%] |
|  | Q2 | 4067 | 46.6% | [45.1%, 48.1%] |
|  | Q3 | 4099 | 46.6% | [45.1%, 48.1%] |
|  | Q4 | 4105 | 46.6% | [45.1%, 48.1%] |
| 2019 | Q1 | 4093 | 46.8% | [45.3%, 48.3%] |
|  | Q2 | 4181 | 46.7% | [45.2%, 48.2%] |
|  | Q3 | 4259 | 46.2% | [44.7%, 47.7%] |
|  | Q4 | 4267 | 46.4% | [44.9%, 47.9%] |
| 2020 | Q1 | 4268 | 45.6% | [44.1%, 47.1%] |
|  | Q2 | 4303 | 45.2% | [43.7%, 46.7%] |
|  | Q3 | 4182 | 45.4% | [43.9%, 46.9%] |
|  | Q4 | 4358 | 44.8% | [43.3%, 46.3%] |
| 2021 | Q1 | 4279 | 44.4% | [42.9%, 45.9%] |
|  | Q2 | 4415 | 43.9% | [42.4%, 45.4%] |
|  | Q3 | 4495 | 43.6% | [42.2%, 45.1%] |
|  | Q4 | 4497 | 44.0% | [42.6%, 45.5%] |
| 2022 | Q1 | 4463 | 43.6% | [42.2%, 45.1%] |
|  | Q2 | 4578 | 42.7% | [41.3%, 44.1%] |
|  | Q3 | 4624 | 42.3% | [40.9%, 43.7%] |
|  | Q4 | 4648 | 42.1% | [40.7%, 43.5%] |
| 2023 | Q1 | 4662 | 42.7% | [41.3%, 44.1%] |
