# Supplementary material for: Trends in psychotropic drug use in nursing home residents with dementia and their association with regulatory changes: A routine care data analysis from 2016 to 2023
Source: PLoS One. 2026 Nov 4;21(11):e0361903. doi: 10.1371/journal.pone.0361903 (PMC13734491; doi:10.1371/journal.pone.0361903)
Supplement: Supplementary file 2 [file pone.0361903.s002.docx]

**S2 Table. Prevalence of PDPs in total and per category, including only regular prescriptions.**

| Year | Quartile | Total % (95% CI) | Antipsychotics %  (95% CI) | Anxiolytics %  (95% CI) | Hypnotics %  (95% CI) | Antidepressants %  (95% CI) | Anti-dementia drugs % (95% CI) |
| --- | --- | --- | --- | --- | --- | --- | --- |
| 2016 | Q1 | 48.3% (46.7%–49.9%) | 19.8% (18.5%–21.1%) | 11.1% (10.1%–12.1%) | 9.3% (8.4%–10.2%) | 25.1% (23.7%–26.5%) | 7.5% (6.7%–8.3%) |
|  | Q2 | 47.1% (45.5%–48.7%) | 19.5% (18.2%–20.8%) | 11.2% (10.2%–12.2%) | 8.9% (8.0%–9.8%) | 24.2% (22.8%–25.5%) | 7.2% (6.5%–8.1%) |
|  | Q3 | 47.3% (45.7%–48.9%) | 19.7% (18.5%–21.0%) | 11.4% (10.4%–12.4%) | 8.7% (7.9%–9.7%) | 24.0% (22.7%–25.3%) | 7.3% (6.5%–8.2%) |
|  | Q4 | 46.4% (44.9%–48.0%) | 19.6% (18.4%–20.9%) | 10.8% (9.9%–11.8%) | 8.6% (7.8%–9.5%) | 23.4% (22.1%–24.7%) | 7.0% (6.2%–7.8%) |
| 2017 | Q1 | 46.6% (45.1%–48.1%) | 19.8% (18.6%–21.0%) | 11.1% (10.2%–12.2%) | 8.5% (7.7%–9.4%) | 23.3% (22.0%–24.6%) | 6.5% (5.8%–7.3%) |
|  | Q2 | 46.8% (45.3%–48.3%) | 20.0% (18.8%–21.3%) | 11.0% (10.0%–12.0%) | 8.1% (7.3%–9.0%) | 23.2% (22.0%–24.6%) | 6.9% (6.1%–7.7%) |
|  | Q3 | 46.4% (44.9%–47.9%) | 20.1% (18.9%–21.4%) | 11.1% (10.1%–12.1%) | 7.5% (6.7%–8.3%) | 23.5% (22.2%–24.8%) | 6.8% (6.1%–7.6%) |
|  | Q4 | 46.8% (45.3%–48.3%) | 20.7% (19.5%–22.0%) | 10.9% (10.0%–11.9%) | 7.2% (6.4%–8.0%) | 23.8% (22.5%–25.1%) | 6.7% (6.0%–7.5%) |
| 2018 | Q1 | 47.6% (46.0%–49.1%) | 20.7% (19.5%–22.0%) | 11.3% (10.4%–12.3%) | 7.8% (7.0%–8.7%) | 24.3% (23.0%–25.7%) | 6.5% (5.8%–7.3%) |
|  | Q2 | 46.6% (45.1%–48.1%) | 20.6% (19.4%–21.9%) | 11.3% (10.4%–12.3%) | 7.3% (6.5%–8.1%) | 25.0% (23.7%–26.3%) | 6.7% (6.0%–7.5%) |
|  | Q3 | 46.6% (45.1%–48.1%) | 21.5% (20.3%–22.8%) | 11.1% (10.2%–12.1%) | 7.3% (6.6%–8.2%) | 24.7% (23.4%–26.1%) | 6.6% (5.8%–7.4%) |
|  | Q4 | 46.6% (45.1%–48.1%) | 21.3% (20.1%–22.6%) | 11.5% (10.6%–12.6%) | 7.2% (6.4%–8.0%) | 24.8% (23.5%–26.1%) | 6.6% (5.9%–7.4%) |
| 2019 | Q1 | 46.8% (45.3%–48.3%) | 21.5% (20.2%–22.8%) | 11.4% (10.5%–12.4%) | 7.5% (6.7%–8.3%) | 24.3% (23.0%–25.6%) | 6.7% (6.0%–7.6%) |
|  | Q2 | 46.7% (45.2%–48.2%) | 21.4% (20.2%–22.7%) | 11.7% (10.8%–12.8%) | 7.7% (6.9%–8.5%) | 24.0% (22.8%–25.4%) | 6.4% (5.7%–7.1%) |
|  | Q3 | 46.2% (44.7%–47.7%) | 21.7% (20.5%–23.0%) | 11.7% (10.8%–12.7%) | 7.5% (6.7%–8.3%) | 23.5% (22.3%–24.8%) | 5.9% (5.2%–6.7%) |
|  | Q4 | 46.4% (44.9%–47.9%) | 21.9% (20.7%–23.1%) | 11.9% (11.0%–12.9%) | 7.3% (6.6%–8.1%) | 23.9% (22.6%–25.2%) | 6.0% (5.3%–6.7%) |
| 2020 | Q1 | 45.6% (44.1%–47.1%) | 21.8% (20.6%–23.1%) | 11.8% (10.9%–12.8%) | 7.1% (6.4%–8.0%) | 23.4% (22.1%–24.7%) | 5.7% (5.0%–6.4%) |
|  | Q2 | 45.2% (43.7%–46.7%) | 21.3% (20.1%–22.5%) | 11.4% (10.5%–12.4%) | 6.5% (5.8%–7.3%) | 23.2% (22.0%–24.5%) | 5.5% (4.9%–6.2%) |
|  | Q3 | 45.4% (43.9%–46.9%) | 21.7% (20.5%–23.0%) | 11.2% (10.3%–12.2%) | 6.7% (6.0%–7.5%) | 23.4% (22.2%–24.7%) | 5.5% (4.8%–6.2%) |
|  | Q4 | 44.8% (43.4%–46.3%) | 21.5% (20.3%–22.7%) | 11.5% (10.6%–12.5%) | 6.7% (6.0%–7.5%) | 23.1% (21.9%–24.4%) | 5.1% (4.5%–5.8%) |
| 2021 | Q1 | 44.4% (42.9%–45.9%) | 21.2% (20.0%–22.5%) | 11.2% (10.3%–12.2%) | 6.3% (5.6%–7.0%) | 23.4% (22.2%–24.7%) | 4.9% (4.3%–5.6%) |
|  | Q2 | 43.9% (42.4%–45.4%) | 21.1% (20.0%–22.4%) | 11.0% (10.1%–11.9%) | 6.0% (5.4%–6.8%) | 23.2% (22.0%–24.4%) | 4.7% (4.1%–5.4%) |
|  | Q3 | 43.6% (42.2%–45.1%) | 21.0% (19.8%–22.2%) | 10.7% (9.9%–11.7%) | 5.9% (5.2%–6.6%) | 23.3% (22.1%–24.6%) | 4.8% (4.2%–5.4%) |
|  | Q4 | 44.0% (42.6%–45.5%) | 21.0% (19.8%–22.2%) | 10.5% (9.6%–11.4%) | 5.6% (5.0%–6.3%) | 23.2% (22.0%–24.5%) | 4.6% (4.1%–5.3%) |
| 2022 | Q1 | 43.6% (42.2%–45.1%) | 21.5% (20.3%–22.7%) | 10.0% (9.2%–11.0%) | 5.9% (5.2%–6.6%) | 22.9% (21.6%–24.1%) | 4.5% (3.9%–5.2%) |
|  | Q2 | 42.7% (41.3%–44.1%) | 20.8% (19.7%–22.0%) | 9.7% (8.8%–10.5%) | 6.0% (5.4%–6.7%) | 23.0% (21.8%–24.2%) | 4.4% (3.9%–5.0%) |
|  | Q3 | 42.3% (40.9%–43.7%) | 21.2% (20.0%–22.4%) | 9.8% (9.0%–10.7%) | 5.8% (5.2%–6.5%) | 22.3% (21.1%–23.5%) | 4.7% (4.1%–5.3%) |
|  | Q4 | 42.1% (40.7%–43.5%) | 20.9% (19.7%–22.1%) | 9.5% (8.7%–10.4%) | 5.9% (5.2%–6.6%) | 22.2% (21.1%–23.4%) | 4.7% (4.1%–5.3%) |
| 2023 | Q1 | 42.7% (41.3%–44.1%) | 20.9% (19.8%–22.1%) | 9.6% (8.8%–10.5%) | 6.3% (5.7%–7.1%) | 21.8% (20.7%–23.0%) | 5.0% (4.4%–5.6%) |
