# Supplementary material for: Trends in psychotropic drug use in nursing home residents with dementia and their association with regulatory changes: A routine care data analysis from 2016 to 2023
Source: PLoS One. 2026 Nov 4;21(11):e0361903. doi: 10.1371/journal.pone.0361903 (PMC13734491; doi:10.1371/journal.pone.0361903)
Supplement: Supplementary file 3 [file pone.0361903.s003.docx]

**S3 Table. Prevalence of PDPs in total with 95% confidence intervals, including only pro re nata prescriptions.**

| Year | Quartile | Sample | Events | Proportion | 95% CI |
| --- | --- | --- | --- | --- | --- |
| 2016 | Q1 | 3765 | 726 | 19.3% | [18.1%, 20.6%] |
|  | Q2 | 3755 | 721 | 19.2% | [18.0%, 20.5%] |
|  | Q3 | 3904 | 783 | 20.1% | [18.8%, 21.3%] |
|  | Q4 | 3915 | 764 | 19.5% | [18.3%, 20.8%] |
| 2017 | Q1 | 4028 | 827 | 20.5% | [19.3%, 21.8%] |
|  | Q2 | 4042 | 809 | 20.0% | [18.8%, 21.3%] |
|  | Q3 | 4070 | 813 | 20.0% | [18.8%, 21.2%] |
|  | Q4 | 4066 | 814 | 20.0% | [18.8%, 21.3%] |
| 2018 | Q1 | 4024 | 867 | 21.5% | [20.3%, 22.8%] |
|  | Q2 | 4067 | 884 | 21.7% | [20.5%, 23.0%] |
|  | Q3 | 4099 | 906 | 22.1% | [20.9%, 23.4%] |
|  | Q4 | 4105 | 882 | 21.5% | [20.3%, 22.8%] |
| 2019 | Q1 | 4093 | 881 | 21.5% | [20.3%, 22.8%] |
|  | Q2 | 4181 | 885 | 21.2% | [20.0%, 22.4%] |
|  | Q3 | 4259 | 910 | 21.4% | [20.2%, 22.6%] |
|  | Q4 | 4267 | 943 | 22.1% | [20.9%, 23.4%] |
| 2020 | Q1 | 4268 | 943 | 22.1% | [20.9%, 23.4%] |
|  | Q2 | 4303 | 923 | 21.5% | [20.2%, 22.7%] |
|  | Q3 | 4182 | 912 | 21.8% | [20.6%, 23.1%] |
|  | Q4 | 4358 | 935 | 21.5% | [20.3%, 22.7%] |
| 2021 | Q1 | 4279 | 966 | 22.6% | [21.3%, 23.9%] |
|  | Q2 | 4415 | 995 | 22.5% | [21.3%, 23.8%] |
|  | Q3 | 4495 | 955 | 21.2% | [20.1%, 22.5%] |
|  | Q4 | 4497 | 945 | 21.0% | [19.8%, 22.2%] |
| 2022 | Q1 | 4463 | 929 | 20.8% | [19.6%, 22.0%] |
|  | Q2 | 4578 | 910 | 19.9% | [18.7%, 21.1%] |
|  | Q3 | 4624 | 901 | 19.5% | [18.4%, 20.7%] |
|  | Q4 | 4648 | 900 | 19.4% | [18.3%, 20.5%] |
| 2023 | Q1 | 4662 | 882 | 18.9% | [17.8%, 20.1%] |
