# Supplementary material for: Trends in psychotropic drug use in nursing home residents with dementia and their association with regulatory changes: A routine care data analysis from 2016 to 2023
Source: PLoS One. 2026 Nov 4;21(11):e0361903. doi: 10.1371/journal.pone.0361903 (PMC13734491; doi:10.1371/journal.pone.0361903)
Supplement: Supplementary file 4 [file pone.0361903.s004.docx]

**S4 Table. Prevalence of PDPs in total and per category, including only pro re nata prescriptions.**

| Year | Quartile | Total % (95% CI) | Antipsychotics %  (95% CI) | Anxiolytics %  (95% CI) | Hypnotics %  (95% CI) | Antidepressants %  (95% CI) | Anti-dementia drugs % (95% CI) |
| --- | --- | --- | --- | --- | --- | --- | --- |
| 2016 | Q1 | 19.3% (18.1%–20.6%) | 3.7% (3.2%–4.4%) | 13.5% (12.4%–14.6%) | 3.4% (2.9%–4.0%) | 0.1% (0.0%–0.2%) | 0.0% (0.0%–0.1%) |
|  | Q2 | 19.2% (18.0%–20.5%) | 3.5% (3.0%–4.2%) | 14.0% (12.9%–15.1%) | 3.2% (2.7%–3.9%) | 0.1% (0.0%–0.2%) | 0.0% (0.0%–0.1%) |
|  | Q3 | 20.1% (18.8%–21.3%) | 3.2% (2.7%–3.8%) | 14.8% (13.8%–16.0%) | 3.3% (2.8%–3.9%) | 0.1% (0.0%–0.2%) | 0.0% (0.0%–0.1%) |
|  | Q4 | 19.5% (18.3%–20.8%) | 3.5% (3.0%–4.1%) | 14.3% (13.2%–15.4%) | 3.3% (2.8%–3.9%) | 0.0% (0.0%–0.1%) | 0.0% (0.0%–0.1%) |
| 2017 | Q1 | 20.5% (19.3%–21.8%) | 4.2% (3.7%–4.9%) | 14.9% (13.8%–16.0%) | 3.3% (2.7%–3.8%) | 0.0% (0.0%–0.1%) | 0.0% (0.0%–0.1%) |
|  | Q2 | 20.0% (18.8%–21.3%) | 3.7% (3.2%–4.4%) | 14.6% (13.6%–15.8%) | 3.3% (2.8%–3.9%) | 0.0% (0.0%–0.1%) | 0.0% (0.0%–0.1%) |
|  | Q3 | 20.0% (18.8%–21.2%) | 3.7% (3.2%–4.3%) | 14.6% (13.5%–15.7%) | 3.5% (3.0%–4.1%) | 0.0% (0.0%–0.1%) | 0.0% (0.0%–0.1%) |
|  | Q4 | 20.0% (18.8%–21.3%) | 3.5% (3.0%–4.2%) | 14.9% (13.8%–16.0%) | 3.5% (3.0%–4.2%) | 0.0% (0.0%–0.1%) | 0.0% (0.0%–0.1%) |
| 2018 | Q1 | 21.5% (20.3%–22.8%) | 4.0% (3.5%–4.7%) | 15.9% (14.8%–17.0%) | 3.4% (2.9%–4.0%) | 0.0% (0.0%–0.1%) | 0.0% (0.0%–0.1%) |
|  | Q2 | 21.7% (20.5%–23.0%) | 4.0% (3.4%–4.6%) | 16.3% (15.2%–17.4%) | 3.3% (2.8%–3.9%) | 0.0% (0.0%–0.2%) | 0.0% (0.0%–0.1%) |
|  | Q3 | 22.1% (20.9%–23.4%) | 4.1% (3.5%–4.7%) | 16.5% (15.4%–17.6%) | 3.3% (2.8%–3.9%) | 0.0% (0.0%–0.2%) | 0.0% (0.0%–0.1%) |
|  | Q4 | 21.5% (20.3%–22.8%) | 4.0% (3.5%–4.7%) | 15.8% (14.7%–16.9%) | 3.3% (2.8%–3.9%) | 0.1% (0.0%–0.2%) | 0.0% (0.0%–0.1%) |
| 2019 | Q1 | 21.5% (20.3%–22.8%) | 4.2% (3.6%–4.9%) | 15.6% (14.6%–16.8%) | 3.2% (2.7%–3.8%) | 0.1% (0.0%–0.3%) | 0.0% (0.0%–0.1%) |
|  | Q2 | 21.2% (20.0%–22.4%) | 4.5% (4.0%–5.2%) | 15.2% (14.1%–16.3%) | 3.2% (2.7%–3.8%) | 0.1% (0.0%–0.2%) | 0.0% (0.0%–0.1%) |
|  | Q3 | 21.4% (20.2%–22.6%) | 4.6% (4.0%–5.2%) | 15.7% (14.7%–16.9%) | 2.8% (2.3%–3.3%) | 0.0% (0.0%–0.2%) | 0.0% (0.0%–0.1%) |
|  | Q4 | 22.1% (20.9%–23.4%) | 4.6% (4.0%–5.3%) | 16.6% (15.5%–17.7%) | 2.6% (2.2%–3.1%) | 0.0% (0.0%–0.1%) | 0.0% (0.0%–0.1%) |
| 2020 | Q1 | 22.1% (20.9%–23.4%) | 4.8% (4.2%–5.5%) | 16.5% (15.4%–17.7%) | 2.6% (2.2%–3.1%) | 0.1% (0.0%–0.2%) | 0.0% (0.0%–0.1%) |
|  | Q2 | 21.5% (20.2%–22.7%) | 4.3% (3.7%–4.9%) | 16.1% (15.0%–17.2%) | 2.7% (2.3%–3.2%) | 0.0% (0.0%–0.2%) | 0.0% (0.0%–0.1%) |
|  | Q3 | 21.8% (20.6%–23.1%) | 4.1% (3.6%–4.8%) | 16.7% (15.6%–17.9%) | 2.9% (2.4%–3.4%) | 0.0% (0.0%–0.2%) | 0.0% (0.0%–0.1%) |
|  | Q4 | 21.5% (20.3%–22.7%) | 4.1% (3.5%–4.7%) | 16.2% (15.2%–17.4%) | 2.7% (2.3%–3.2%) | 0.1% (0.0%–0.2%) | 0.0% (0.0%–0.1%) |
| 2021 | Q1 | 22.6% (21.3%–23.9%) | 4.4% (3.8%–5.0%) | 17.2% (16.1%–18.4%) | 2.5% (2.0%–3.0%) | 0.1% (0.0%–0.2%) | 0.0% (0.0%–0.1%) |
|  | Q2 | 22.5% (21.3%–23.8%) | 4.1% (3.6%–4.7%) | 17.7% (16.6%–18.8%) | 2.2% (1.8%–2.6%) | 0.0% (0.0%–0.1%) | 0.0% (0.0%–0.1%) |
|  | Q3 | 21.2% (20.1%–22.5%) | 4.0% (3.4%–4.6%) | 16.8% (15.7%–17.9%) | 2.3% (1.9%–2.7%) | 0.0% (0.0%–0.2%) | 0.0% (0.0%–0.1%) |
|  | Q4 | 21.0% (19.8%–22.2%) | 3.9% (3.4%–4.5%) | 16.4% (15.4%–17.5%) | 2.1% (1.7%–2.5%) | 0.0% (0.0%–0.1%) | 0.0% (0.0%–0.1%) |
| 2022 | Q1 | 20.8% (19.6%–22.0%) | 3.9% (3.4%–4.5%) | 16.0% (15.0%–17.1%) | 2.3% (1.9%–2.7%) | 0.0% (0.0%–0.1%) | 0.0% (0.0%–0.1%) |
|  | Q2 | 19.9% (18.7%–21.1%) | 4.0% (3.5%–4.6%) | 15.3% (14.3%–16.4%) | 2.1% (1.8%–2.6%) | 0.0% (0.0%–0.1%) | 0.0% (0.0%–0.1%) |
|  | Q3 | 19.5% (18.4%–20.7%) | 3.7% (3.2%–4.2%) | 15.2% (14.2%–16.3%) | 2.0% (1.6%–2.5%) | 0.0% (0.0%–0.1%) | 0.0% (0.0%–0.1%) |
|  | Q4 | 19.4% (18.3%–20.5%) | 3.6% (3.1%–4.1%) | 15.0% (14.0%–16.0%) | 2.1% (1.7%–2.5%) | 0.0% (0.0%–0.1%) | 0.0% (0.0%–0.1%) |
| 2023 | Q1 | 18.9% (17.8%–20.1%) | 3.4% (2.9%–4.0%) | 14.9% (13.9%–15.9%) | 1.9% (1.6%–2.4%) | 0.0% (0.0%–0.1%) | 0.0% (0.0%–0.1%) |
