# Supplementary material for: Trends in psychotropic drug use in nursing home residents with dementia and their association with regulatory changes: A routine care data analysis from 2016 to 2023
Source: PLoS One. 2026 Nov 4;21(11):e0361903. doi: 10.1371/journal.pone.0361903 (PMC13734491; doi:10.1371/journal.pone.0361903)
Supplement: Supplementary file 5 [file pone.0361903.s005.docx]

**S5 Table. Prevalence of anti-convulsant prescriptions in total with 95% confidence intervals, including only regular prescriptions.**

| **Year** | **Quartile** | **Sample** | **Proportion** | **95% CI** |
| --- | --- | --- | --- | --- |
| **2016** | Q1 | 3765 | 4.6% | [4.0%, 5.3%] |
|  | Q2 | 3755 | 4.3% | [3.7%, 5.0%] |
|  | Q3 | 3904 | 4.4% | [3.8%, 5.1%] |
|  | Q4 | 3915 | 4.4% | [3.8%, 5.1%] |
| **2017** | Q1 | 4028 | 4.3% | [3.7%, 5.0%] |
|  | Q2 | 4042 | 4.4% | [3.8%, 5.0%] |
|  | Q3 | 4070 | 4.4% | [3.8%, 5.0%] |
|  | Q4 | 4066 | 4.4% | [3.8%, 5.1%] |
| **2018** | Q1 | 4024 | 4.6% | [4.0%, 5.3%] |
|  | Q2 | 4067 | 5.0% | [4.3%, 5.7%] |
|  | Q3 | 4099 | 5.1% | [4.4%, 5.8%] |
|  | Q4 | 4105 | 5.0% | [4.4%, 5.7%] |
| **2019** | Q1 | 4093 | 5.1% | [4.4%, 5.8%] |
|  | Q2 | 4181 | 5.1% | [4.5%, 5.9%] |
|  | Q3 | 4259 | 5.3% | [4.7%, 6.0%] |
|  | Q4 | 4267 | 5.5% | [4.9%, 6.3%] |
| **2020** | Q1 | 4268 | 5.4% | [4.8%, 6.2%] |
|  | Q2 | 4303 | 5.2% | [4.6%, 5.9%] |
|  | Q3 | 4182 | 4.9% | [4.3%, 5.6%] |
|  | Q4 | 4358 | 4.6% | [4.0%, 5.3%] |
| **2021** | Q1 | 4279 | 4.6% | [4.0%, 5.3%] |
|  | Q2 | 4415 | 4.8% | [4.2%, 5.4%] |
|  | Q3 | 4495 | 4.9% | [4.3%, 5.5%] |
|  | Q4 | 4497 | 5.0% | [4.4%, 5.7%] |
| **2022** | Q1 | 4463 | 5.2% | [4.6%, 5.9%] |
|  | Q2 | 4578 | 5.1% | [4.5%, 5.8%] |
|  | Q3 | 4624 | 5.2% | [4.6%, 5.9%] |
|  | Q4 | 4648 | 5.0% | [4.4%, 5.6%] |
| **2023** | Q1 | 4662 | 4.8% | [4.2%, 5.4%] |
